# Editorial: Peer feedback in second/foreign language writing classrooms: educational psychology perspective

**DOI:** 10.3389/fpsyg.2023.1266437

**Published:** 2023-08-25

**Authors:** Xinghua Liu, Rui Alexandre Alves, Josef Schmied, Angelique Aitken

**Affiliations:** ^1^School of Foreign Languages, Shanghai Jiao Tong University, Shanghai, China; ^2^Faculty of Psychology and Education Sciences, University of Porto, Porto, Portugal; ^3^English Department, Chemnitz University of Technology, Chemnitz, Germany; ^4^Department of Educational Psychology, The Pennsylvania State University, University Park, PA, United States

**Keywords:** peer feedback in classroom, peer feedback activity, peer feedback literacy, cognitive complexity, rubric development

Peer feedback (hereafter known as PF) has long been practiced and researched over the past decades, and has been termed variously by different scholars under such names as “peer review,” “peer editing,” “peer evaluation,” or “peer response.” In this Research Topic, we define PF as the practice of second/foreign language learners (ESL/EFL) assuming responsibilities in providing feedback upon peer's written drafts in either writing and/or oral formats during the process of writing. PF research has been gaining momentum in recent years and has been increasingly popular in ESL/EFL writing classrooms due to its instructional values and learning potentials. Mounting evidence tends to show that PF activities may be beneficious not only for learning domain knowledge such as students' better language performance, but also for fostering those skills like reader awareness, motivation and self-efficacy, reflective and critical thinking.

However, we find that existing PF research has predominantly centered on a linguistic perspective. For example, previous studies largely revolved around topics like inappropriate language usages student reviewers could identify, language use in feedbacking forward and backward, and the types of revisions students would conduct. Hence, the purpose of this Research Topic is to gather research that examines PF from an educational psychology perspective with a particular focus on topics like teachers and students' attitudes, effects of different modes of delivery, and individual differences in PF. There are six papers in this Research Topic and we are going to introduce them briefly.

Zhang X. S. et al. conducted an interview-based research examining five Chinese university EFL writing teachers' attitude toward student self-assessment of writing and their corresponding self-efficacy beliefs during the process. It was found that on one hand, teachers recognized the positive effects of self-assessment upon students' learning, but on the other hand, they still preferred the traditional teacher-controlled assessments. In terms of their confidence to implement self-assessment of writing, all teachers rated themselves relatively low in self-efficacy levels.

Based on the experiences of 12 doctoral students in an academic writing course in a Chinese university, Zhang M. et al. explored how different online platforms (i.e., Moodle, WeChat, Rain Classroom) can work together effectively to deliver PF. The study found that working on the three different platforms was overall useful for students to revise their academic writing work. The study also identified various emotions and affect students had while participating in online PF. At the pedagogical level, the authors offered practical suggestions on how to maximize different technical platforms to better address students' social-affective needs during the PF process.

Cao et al. did a systematic review study to explore the benefits of using online PF in ESL/EFL writing. They found that overall ESL/EFL students had positive attitudes toward the online PF practice. Compared to face-to-face PF, online PF could mitigate face embarrassment and the effects of writing anxiety, thus enhancing writing motivation; generate more revision-based comments and help improve writing performance; build up a better learning environment and accelerate students' reflection, critical thinking and responsibility.

Wu et al. conducted a quasi-experimental study to examine the effects of providing PF to other students' writings upon their genre awareness in business letter writing. Students in the experimental group reviewed the other students' drafts at different levels (high, medium, and low) and gave written comments. Students in the control group did not receive treatment but instead did self-revision. It was found that PF together with weakness feedback comments for medium-level peers' drafts contributed positively to learners' genre awareness in business letter writing.

Sun et al. compared PF to teacher feedback and explored the different features of feedback and its impact on draft revisions. Overall, they found that PF could supplement teacher feedback, alter some linguistic characteristics and may lead to higher scores. Specifically, it was found that PF was helpful with text cohesion and the lexical quality of writing, but it was not so helpful in syntactic complexity of the essay. The authors also pointed out that though students could identify many issues in writing, they couldn't provide sufficient or useful explanations, solutions or suggestions compared to the teacher.

By adopting engagement with task framework, Chen et al. examined the cognitive, behavioral, social, and affective dimensions of engagement in a collaborative task. It was found that the aforementioned four dimensions were interwoven and interdependent. For example, those who showed high social engagement and acknowledged benefits of peer collaboration were likely to be increasingly engaged with the task in the cognitive and behavioral dimensions. By contrast, negative emotions and perceptions of the task would result in a withdrawal from interaction.

The six papers in this Research Topic are telling about current invaluable research on PF. In the future, we hope more attention could be given to the following two areas of studies. First, the cognitive processes of giving and taking feedback have been largely unknown. For instance, we do not know clearly the planning and decision-making strategies students take while giving or taking PF. Second, the design and validation of effective PF training, PF rubrics and the assessment methods of PF performance have been under-researched. Research in these two areas is crucial and will surely add to forthcoming Research Topics.

## Author contributions

XL: Conceptualization, Writing—original draft. RA: Conceptualization, Writing—original draft, Writing—review and editing. JS: Conceptualization, Writing—original draft, Writing—review and editing. AA: Conceptualization, Writing—original draft, Writing—review and editing.

